# Hot Carrier Cooling and Trapping in Atomically Thin WS_2_ Probed by Three-Pulse Femtosecond Spectroscopy

**DOI:** 10.1021/acsnano.2c10479

**Published:** 2023-03-20

**Authors:** Tong Wang, Thomas R. Hopper, Navendu Mondal, Sihui Liu, Chengning Yao, Xijia Zheng, Felice Torrisi, Artem A. Bakulin

**Affiliations:** † Department of Chemistry and Centre for Processable Electronics, Imperial College London, London W12 0BZ, United Kingdom; ‡ Department of Materials Science and Engineering, Stanford University, Stanford, California 94305, United States; § Dipartimento di Fisica e Astronomia, Universita’ di Catania & CNR-IMM (Catania Universita’), Via S. Sofia 64, 95123 Catania, Italy

**Keywords:** atomically thin 2D materials, ultrafast spectroscopy, hot carrier cooling, hot-phonon bottleneck, hot carrier trapping

## Abstract

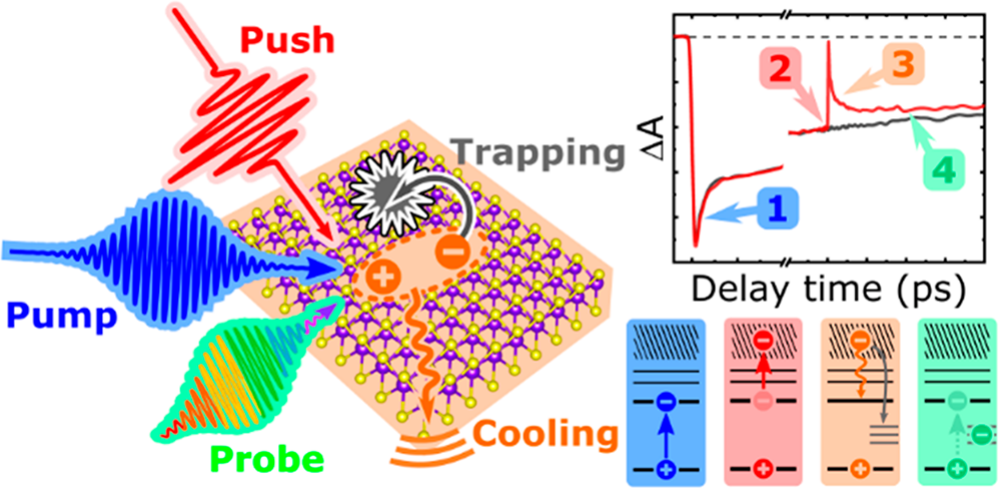

Transition metal dichalcogenides (TMDs) have shown outstanding semiconducting properties which make them promising materials for next-generation optoelectronic and electronic devices. These properties are imparted by fundamental carrier–carrier and carrier–phonon interactions that are foundational to hot carrier cooling. Recent transient absorption studies have reported ultrafast time scales for carrier cooling in TMDs that can be slowed at high excitation densities via a hot-phonon bottleneck (HPB) and discussed these findings in the light of optoelectronic applications. However, quantitative descriptions of the HPB in TMDs, including details of the electron–lattice coupling and how cooling is affected by the redistribution of energy between carriers, are still lacking. Here, we use femtosecond pump–push–probe spectroscopy as a single approach to systematically characterize the scattering of hot carriers with optical phonons, cold carriers, and defects in a benchmark TMD monolayer of polycrystalline WS_2_. By controlling the interband pump and intraband push excitations, we observe, in real-time (i) an extremely rapid “intrinsic” cooling rate of ∼18 ± 2.7 eV/ps, which can be slowed with increasing hot carrier density, (ii) the deprecation of this HPB at elevated cold carrier densities, exposing a previously undisclosed role of the carrier–carrier interactions in mediating cooling, and (iii) the interception of high energy hot carriers on the subpicosecond time scale by lattice defects, which may account for the lower photoluminescence yield of TMDs when excited above band gap.

Two-dimensional (2D) transition metal dichalcogenides (TMDs) are widely recognized as promising materials for next-generation ultrathin, flexible optoelectronic devices.^1−4^ Compared to their bulk or multilayer counterparts, atomically thin single layer (SL) TMDs are of particular interest and have been successfully integrated into a plethora of devices such as light-emitting diodes,^5,6^ photovoltaics,^7−9^ and spintronics^10^ that leverage their direct band gaps,^11,12^ strong spin–orbital coupling,^13,14^ high exciton binding energy,^15−17^ and intriguing spin-valley physics.^18,19^ These properties are intimately linked to the relaxation of charge carriers, and more broadly to the flow of energy between charge carriers, the lattice, and the defects therein. Despite several attempts to understand how charge carriers relax and interact with defects in TMDs, these processes remain only partially understood, hindering the optimization and reproducibility of devices.^20−22^

As in other semiconductors, “hot” electrons (holes) with initial kinetic energy above the conduction (valence) band edges can be generated in SL TMDs by above-gap optical or electrical injection of charges, or high-order Auger-type recombination processes. The formation and subsequent fate of the hot carriers in semiconductor nanomaterials have been interrogated by a variety of means, though most ubiquitously by pump–probe techniques employing ultrashort laser pulses.^20,23−26^ In 2D TMDs, above-gap optical excitation generates free hot carriers with a high transient electronic temperature of thousands of Kelvin.^27,28^ The excess energy of the free hot carriers is dissipated to the lattice via the coupling to longitudinal optical (LO) phonons and results in a slightly elevated lattice temperature^21,29^ and “cold” bound excitons.^20,30^ The cooling process occurs mostly on the picosecond time scale, but it can be further slowed down due to a hot-phonon bottleneck (HPB) effect.^20,31^ The HPB effect typically occurs when excess energy released from a large density of hot carriers is dissipated into LO phonons. The hot phonons are reabsorbed by the carriers to keep the electronic bath hot, prolonging the overall cooling process.^32^ The slowed cooling allows for the hot carriers to be directly extracted at material heterojunctions^33−35^ or participate in carrier multiplication,^36,37^ which are both highly desirable for efficient photon-to-electron conversion.

The early time transient absorption (TA) response is commonly used to investigate the time scale for cooling in semiconductors. However, many studies have shown that early time TA response for SL and/or multilayer (ML) TMDs involves multiple overlapping processes, including: Auger-type exciton–exciton annihilation,^38−40^ exciton formation and dissociation,^41,42^ state filling,^43−45^ ultrafast trapping,^46,47^ and band gap renormalization.^48,49^ Critically, the number of hot carriers cannot be systematically controlled without also inducing these many-body effects. For instance, Wang et al. found that with increasing pump intensity, the HPB and Auger heating effect can contribute simultaneously to prolong the carrier relaxation process in SL-MoS_2_.^26^ The upshot of this is that conventional two-pulse pump–probe (PP) spectroscopy cannot always isolate the hot carrier dynamics in TMDs from the other phenomena occurring in the ultrafast time domain by solely controlling the pump parameters.

Recently, we implemented PPP spectroscopy to study hot carrier cooling dynamics in halide perovskites^50^ and nanocrystals.^51,52^ In this technique, an intense time-delayed near infrared (NIR) push pulse is used to optically heat the cold band-edge carriers produced by an initial interband pump pulse.^53−56^ The time delay between the excitation pump and heating push is tunable by mechanical control of the beam path lengths. Therefore, the energy and population of hot carriers can be selectively controlled, and the push-induced effect can be separated from the many-body interactions that occur immediately after the initial pump excitation. By changing the pump fluence, the effect of the initial cold carrier density can also be investigated.^51^ For example, we have demonstrated that in perovskite-based materials, an increased cold carrier density can accelerate carrier cooling through the more rapid dissipation of excess energy via carrier–carrier scattering compared to the HPB effect.^51^

Herein, we utilize three-pulse visible pump–NIR push–visible probe spectroscopy to systematically study the behavior of hot carriers in polycrystalline SL-WS_2_ as a function of their population and energy. With increasing hot carrier density, we observe a slowing of the carrier cooling. This agrees with previous TA spectroscopy studies on TMDs and other low-dimensional materials that exhibit a HPB.^26,34,57,58^ The precise control of the hot carrier density allows us to separate the effect of this phenomenon from an “intrinsic” density-independent carrier cooling rate of ∼18 ± 2.7 eV/ps in SL-WS_2._ Meanwhile, by controlling the number of cold carriers in the system, we discover that the slowed cooling effect by the HPB is diminished by an increase of the cold carrier density. This underscores that carrier–carrier coupling plays a direct role in determining the rate of carrier relaxation in TMDs and that this should not be neglected when considering the ultrafast photophysics of these materials. Adjusting the energy of the hot carriers in our three-pulse technique also allows us to unveil evidence of trapping during the hot carrier cooling process in SL-WS_2_, which provides an additional explanation for the lower photoluminescent quantum yield when TMD materials are excited with high energy photons.^59^ We summarize the work by proposing a fully quantitative model of hot carrier cooling dynamics in TMDs based on experimental observations.

## Results and Discussion

**Figure 1:**
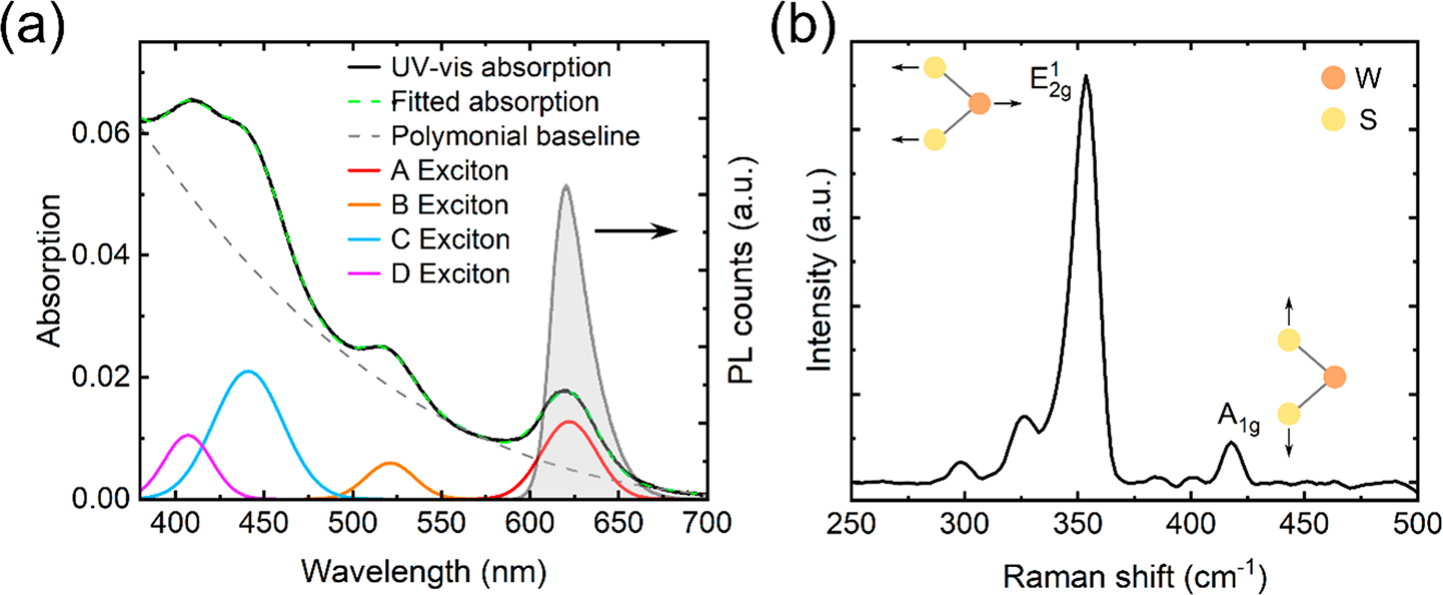
Linear optical and vibrational spectra of SL-WS_2_. (a) UV–vis absorption (solid dark curve) and PL spectra (gray shaded region, excited at 532 nm). The dashed green curved shows the fitting of the absorption spectra by a polynomial baseline (dashed gray curve) and four Gaussian peaks, corresponding to the A, B, C, and D exciton resonances (red, orange, blue, and magenta, respectively). (b) Raman spectrum of SL-WS_2_ with 532 nm excitation, with depictions of the in-plane and out-of-plane vibrational modes.

In this study, we perform experiments on a commercially procured WS_2_ SL grown by chemical vapor deposition and transferred to a quartz substrate as a benchmark TMD system. The steady-state UV–vis absorption spectrum is shown in Figure 1a. This spectrum can be reproduced by four Gaussian peaks and a second-order polynomial baseline. Based on our fitting, the A, B, C, and D excitonic resonances are situated at 620, 520, 440, and 407 nm, respectively, which agree well with the literature.^60,61^ In momentum space, the A and B resonances originate from transitions at the Κ and Κ′ points of the Brillouin Zone, with energy spacing defined by the splitting of the valence band by spin–orbit coupling.^15,26,60^ Meanwhile, the C exciton is understood to arise from the band nesting region where the valence and conduction bands are parallel over an extended region between the Γ and Λ points. The even higher lying D exciton is thought to reside close to the Γ point outside the band nesting region.^60^

The PL spectrum from the SL-WS_2_ in Figure 1a contains a single peak at around 620 nm, which originates from the radiative recombination of the lowest-lying A exciton. The asymmetry of the spectrum has been ascribed to the presence of defects in polycrystalline films.^62^Figure 1b shows the Raman spectrum of the SL-WS_2_ upon excitation at the same wavelength (532 nm). The difference (Δω) between the E^1^_2g_ mode (∼355 cm^–1^, in-plane vibration) and A_1g_ mode (∼417 cm^–1^, out-of-plane vibration) is approximately 62 cm^–1^. These values are consistent with previous Raman spectroscopy characterizations of SL-WS_2_.^62^ We further characterize the SL nature and ∼0.86 nm thickness of the WS_2_ sample by atomic force microscopy (AFM), presented in Figure S1.^63,64^

**Figure 2:**
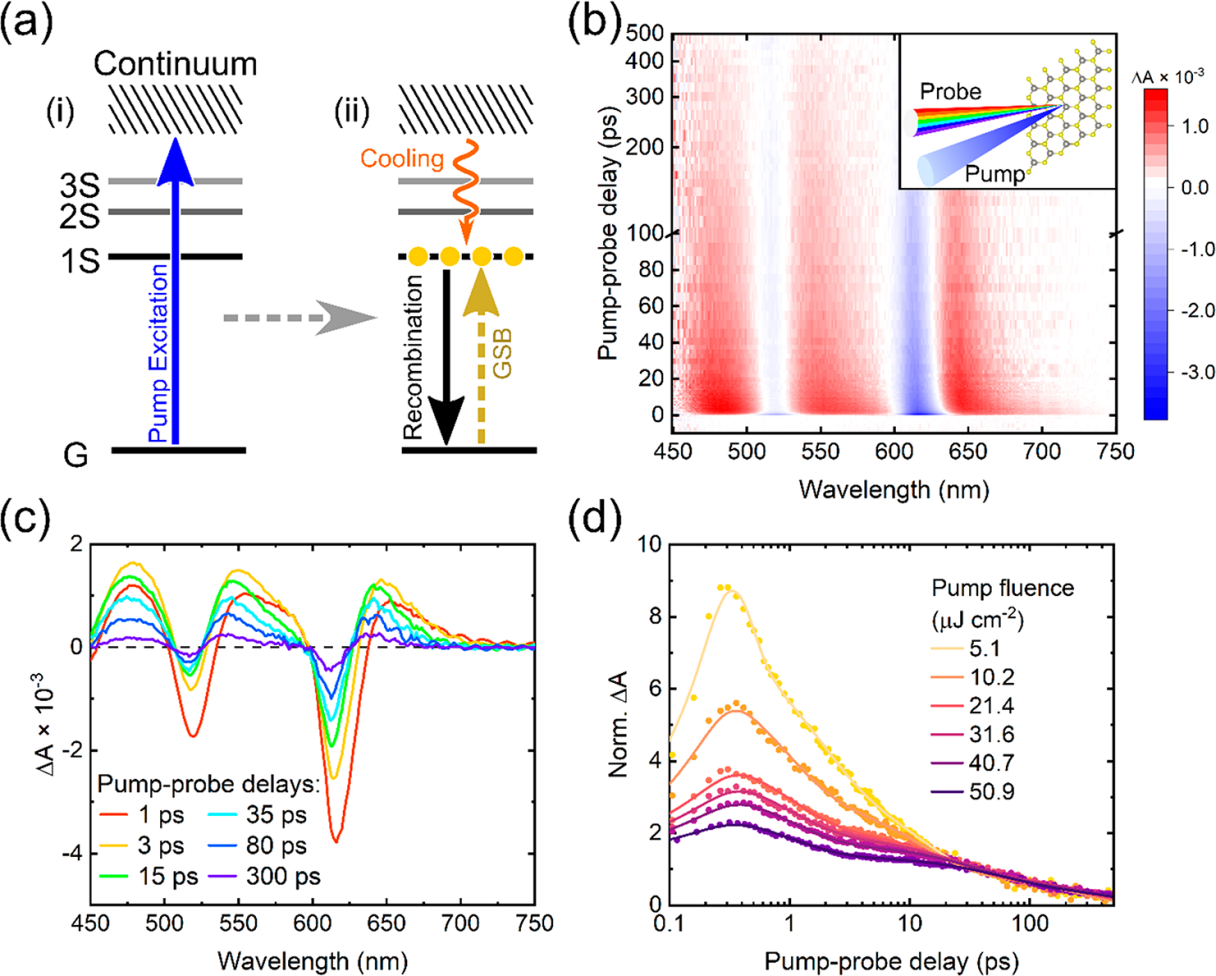
Pump–probe spectroscopy of SL-WS_2_. (a) State diagram depicting the action of the pulses in the PP technique. (b) and (c) TA heatmap and corresponding spectra at time slices for SL-WS_2_ excited by a 400 nm pump (3.1 eV, 21.7 μJ cm^–2^). (d) Kinetics of the A exciton GSB at different pump fluences, normalized by the signal amplitudes at 40 ps. The solid lines are fits by a Gaussian convoluted multiexponential decay.

To study the charge dynamics in SL-WS_2_, we perform complementary two- and three-pulse TA experiments. The diagram for the full setup is presented in Figure S2. In the former PP case, a 400 nm pump is used to excite electron–hole pairs across the band gap, and a broadband probe spanning the visible region is used to probe the resultant charge dynamics. The action of the pulses are depicted in steps (i) and (ii) of Figure 2a, alongside the TA surface and corresponding time-resolved spectral slices in Figures 2b,c. Due to Pauli blocking, the conduction band minimum (or valence band maximum) is filled by excited electrons (or holes) after photoexcitation by the pump, and thus, the absorption at around the band gap is reduced. This reduced absorption (negative signal, shown in blue) produces a ground state bleach (GSB). The high-contrast features with negative amplitude at around 620 and 520 nm are assigned to the GSB of the A and B exciton resonances identified in the linear absorption spectra.^16,41^ We expect similar GSB features from the higher-lying C and D exciton bands, but these fall outside the bandwidth of our visible probe.

The three positive Δ*A* regions in Figure 2b (shown in red) at ∼670, 570, and 470 nm are assigned as photoinduced absorption (PIA) from the A, B, and C excitons, respectively. Within the first 10 ps, we observe a time-dependent ∼3 nm blueshift of both the A and B PIA features that have previously been attributed to the relaxation of hot carriers generated by the above-gap pump, band gap renormalization,^41,60^ and other excited-state transitions.^41,65^ As the origin of these PIA features and their assignment to any direct excitonic transitions remain disputed, we relied on the GSB peaks as a marker for the charge dynamics in this study. Although it should be noted that GSB peaks can represent a mixture of different phenomena, such as the Stark effect and band gap renormalization, the nature of the excited-state transition in GSB peaks is easier to interpret. GSB comes from depopulation of the ground state and, therefore, all the excitons A, B, and C contribute to it in the same way.

To study the excited state population dynamics further, we conducted measurements at different excitation fluences. The normalized transients of the A exciton GSB are shown in Figure 2d. The later-time dynamics (>40 ps) are independent of the excitation density, and the Δ*A* amplitude in this time window scales linearly with the pump fluence (Figure S3). We assign this behavior to monomolecular recombination of the A-exciton, ostensibly due to radiative recombination.^66^ Meanwhile, the early time dynamics (<40 ps) are dependent on the excitation density, and the Δ*A* amplitude scales nonlinearly with the pump fluence (Figure S3). Based on the transients alone, we cannot confidently point to a single mechanism at the heart of this behavior. Previous reports invoke trapping effects and many-body interactions such as Auger recombination, exciton–exciton annihilation, or band gap renormalization.^39,48,67−69^ This serves to demonstrate that it is often complicated to extract the hot carrier cooling dynamics from the early time TA-traces in two-pulse experiments.

**Figure 3:**
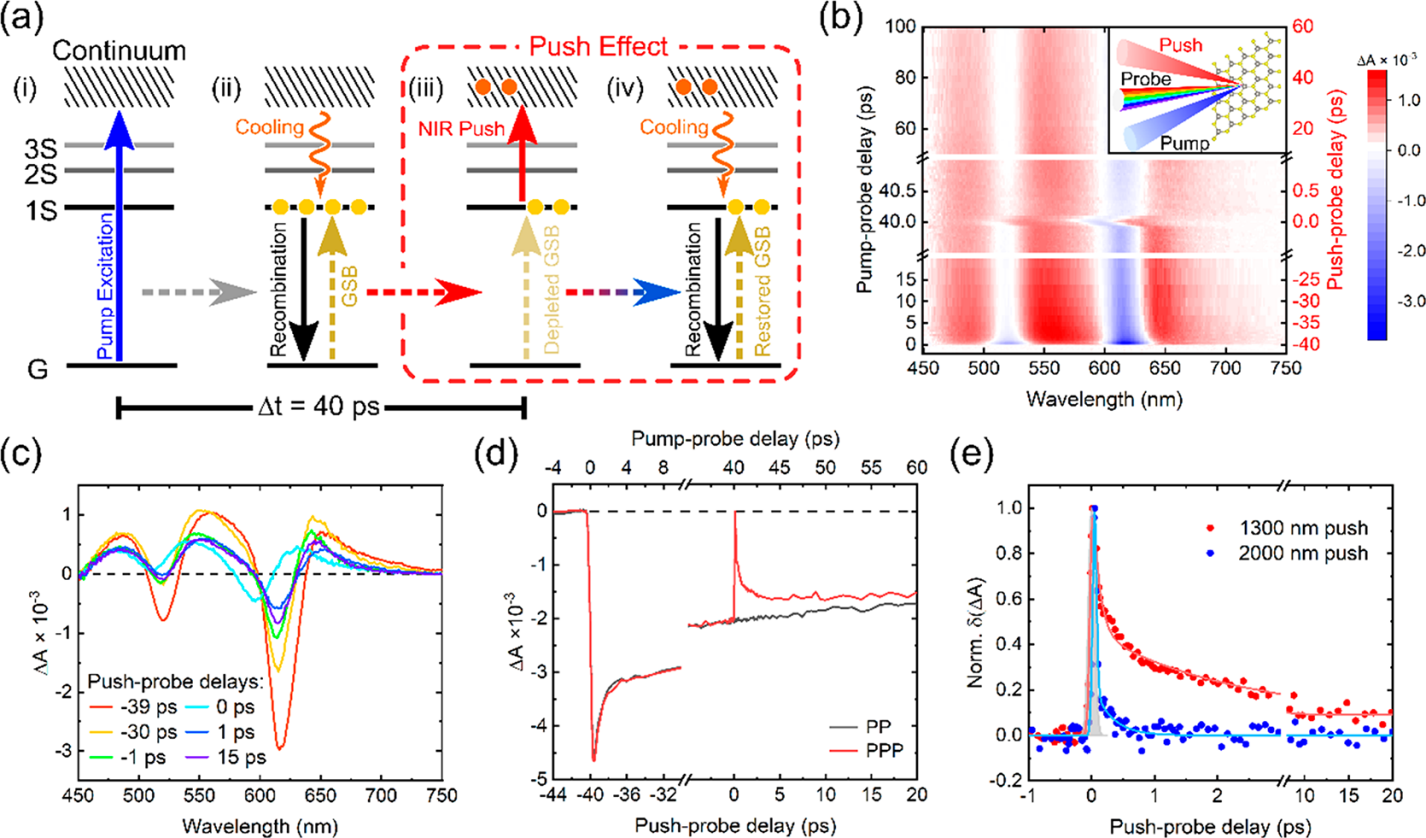
Pump–push–probe spectroscopy of SL-WS_2_. (a) State diagram depicting the action of the pulses in the PPP technique. (i) and (ii) Carrier transitions which are observed in the PP technique. (iii) A time-delayed NIR push pulse (40 ps after the pump) is introduced to heat up the cold states to hot carriers, resulting in reduced GSB peak amplitude. As the carriers cool, the reduced GSB peak amplitude recovers to the stronger PP amplitude without push, shown in (iv). (b) and (c) TA heatmap and corresponding spectra at time slices for SL-WS_2_ with the same 400 nm pump excitation (3.1 eV, 21.7 μJ cm^–2^) as the PP measurement, but with the 1300 nm push (0.95 eV, 9.2 mJ cm^–2^) on. (d) Kinetics of the A exciton GSB with the 1300 nm push off (PP, black) and on (PPP, red). (e) Push-induced δ(Δ*A*) kinetics obtained by Δ*A*_PPP_ – Δ*A*_PP_ for 1300 nm push (in red, 0.95 eV, 9.2 mJ cm^–2^) and 2000 nm push (in blue, 0.62 eV, 10.7 mJ cm^–2^). The solid lines are the Gaussian convoluted multiexponential fits.

To avoid this ambiguity, we designed a PPP experiment with NIR beam that is delayed with respect to the initial pump, offering better control of key experimental parameters, including the density of cold and hot carriers. This allows us to extract not only the cooling time scale, but also critical information about the interactions of hot carriers with cold carriers and defects in a way that conventional PP experiments cannot. Figure 3a depicts the action of the pulses in the PPP scheme. Given the highest pump fluences we used in this study (21.7 μJ cm^–2^) and the absorption cross-section extracted from Figure 1a, the initial carrier density after interband excitation, *n*_0_^*total*^, in the SL-WS_2_ film is estimated to be ∼10^12^ cm^–2^ by eq S1, which is well below the reported Mott density of SL-WS_2_ (1.1 × 10^14^ cm^–2^) where excitons transition to an electron–hole plasma.^16^ Therefore, the cold excited states acted upon by the NIR push can be treated as excitons rather than free carriers or polarons.

Based on the Rydberg model for excitons in TMDs, we assume that the cold excitons are promoted to the band continua by the NIR push, with excess energy determined by the push photon wavelength.^15,43^ Within this picture, carrier cooling refers to the relaxation of hot carriers in the continuum to the cold bound electron–hole state where the constituent charges reside at the band edges. Figure S4 confirms the transition from the exciton to the continuum in photoexcited SL-WS_2_. Based on this broad NIR absorption feature, we chose two push wavelengths at 1300 or 2000 nm to give the cold states appreciable excess energies of 0.95 or 0.62 eV, respectively. To further ensure that the NIR push interacts exclusively with the cold states, we delay the push by 40 ps with respect to the pump so that initial hot carriers generated by the above-band-gap pump excitation relax to the band-edge state and the NIR push pulse does not interact with many-body processes. As seen in Figure 2d, the TA response of the relatively long-lived pump induced states are not determined by the pump fluence in this instance of time, but the TA response is still tractable to allow any small push-induced changes to be measured precisely. We presume that when the NIR push pulse arrives, a portion of the cold carriers are heated up, partially depopulating the conduction or valence band edges to higher energy states, as depicted in step (iii) of Figure 3a. This leads to a transient reduction in the amplitude of the GSB, which recovers when the hot carriers cool, depicted in step (iv).

Figure 3b plots the spectrally resolved data as a function of the push-probe delay time. The spectral features are the same as in the PP experiment in Figure 2, except for a blueshift of the exciton resonances which we ascribe to the optical Stark effect (OSE), as discussed in further detail later. Figure 3c shows that the GSB peak amplitudes for both the A and B excitons are reduced upon the arrival of the push and only partially recover. This indicates that the NIR push pulse provides excess energy to the band edge cold carriers. Although the NIR push pulses are intense, we do not observe the effect of multiphoton absorption. Re-excitation of the sample by the NIR push pulse would produce a more intense GSB signal, rather than the decrease observed in Figure 3d, which compares the PPP and PP dynamics of the A exciton.

To analyze the push effect specifically, we calculated the difference between PPP and PP signals, for two different push wavelengths of 1300 and 2000 nm. The resulting δ(Δ*A*) kinetics in Figure 3e are comprised of three components: (1) a very fast ∼100 fs component, (2) a few-ps component dominating the recovery, and (3) a minor long-lived component with >10 ps decay time for the 1300 nm push, but not for the 2000 nm push.

**Figure 4:**
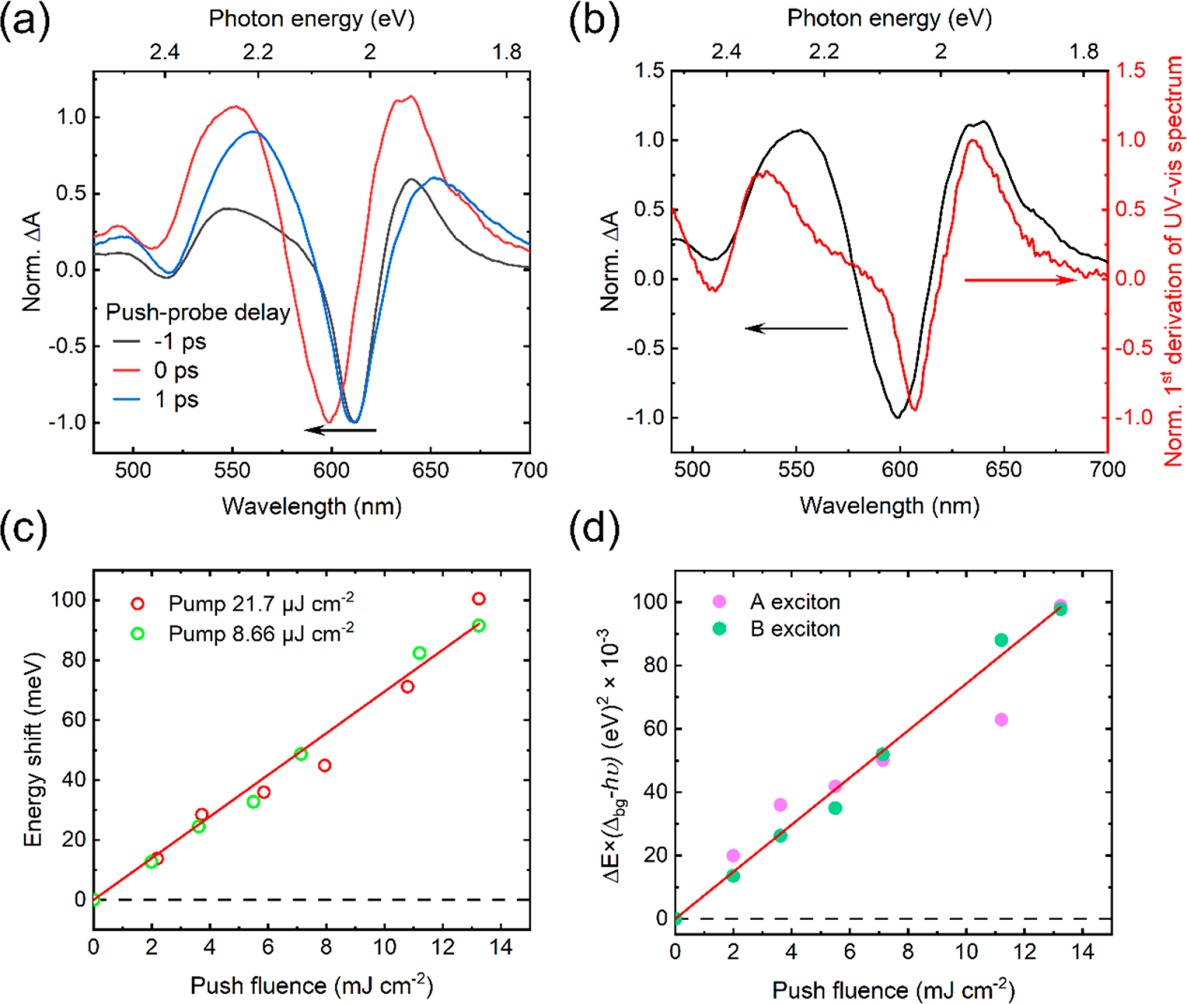
Optical Stark effect upon the optical heating of carriers in SL-WS_2_. (a) Normalized PPP spectra around the A exciton GSB at −1 ps (before push), 0 ps (at push), and at 1 ps (after push). The 1300nm push fluence is 7.2 mJ cm^–2^. (b) PPP spectrum at 0 ps (dark line) and the first derivation of the UV–vis absorption spectrum (red line). (c) Push fluence dependence of the maximum Stark shifting for the A exciton GSB (linear fit shown by solid red line). (d) Δ*E* × (Δ_bg_ – *h*ν), for A exciton and B exciton with a linear fitting (solid red line, for B exciton).

The kinetics of the first fast component around 0 ps follow the ∼100 fs instrument response function of our setup, as seen in Figure 3e. This indicates that this component originates from a nonresonant interaction between the push pulse and SL-WS_2_.^70,71^Figure 4a compares PPP spectra of SL-WS_2_ before, after, and during the push arrival, corresponding to the initially cold, hot, and cooled states, respectively. We observe that upon the push pulse arrival (0 ps), the TA spectrum blueshifts by ∼20 nm, producing a line shape that follows the first derivative of the UV–vis absorption spectrum, displayed in Figure 4b. This suggests that the initial (∼100 fs) dynamics stem from the OSE generated by the electric field of the push pulse. Similar OSE shifts have been observed in PPP studies on organic materials^72^ and PP studies on TMDs^70,71^ and other nanomaterials.^73−75^ However, in comparison to single quantum dots and wells, we did not observe significant dispersive spectral lineshapes before the arrival of NIR push pulse (details are shown in the Supporting Information).^76,77^

The Stark energy shift (Δ*E*) in a two-level system can be quantified as
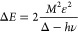
1where ε is the applied electric field, *M* is the polarization matrix element between the initial and final states, Δ*E* is the energy difference between these states, *h* is the Planck constant, and ν is the photon frequency of the incident light.^70,71^ In this work, the initial and final states are the ground state and the first excited state of WS_2_. Therefore, the energy shift is linearly proportional to ε^2^, which is proportional to the push light intensity. Figure 4c demonstrates that our results fit this model well. The maximum shift is only linearly dependent on the push fluence but independent of the pump fluence. Note that the experimental Δ*E* is calculated from the shift in the GSB peak position incurred by the application of the push by comparing the transient spectra at −1 and 0 ps push-probe delay.

Figure 4d compares the OSE on the A and B excitons by presenting their respective Δ*E*(Δ – *hν*) dependences as a function of the push fluence. Based on the shift of the GSB peak wavelengths, the energy difference between the ground state and the first excited state is 2.02 and 2.39 eV for the A and B excitons, respectively. In Figure 4d, the scaling of Δ*E*(Δ – *hν*) with the push fluence does not show much difference for the A and B excitons and obeys the linear fitting well. This implies that the values of *M*^2^ for the A and B excitons are almost identical, which agrees with previous studies of SL-MoS_2_ that indicate that the oscillator strengths of the A and B excitons are similar because they both originate at around the K (or Κ′) valley.^78^ It is worth remarking on the fact that in previous studies of the OSE on TMDs, circularly polarized PP was utilized, and consequently, only the response of A (or B) excitons under an electric field could be investigated due to the valley selection rules in TMDs.^70,71,78^ Herein, we demonstrate that the use of linearly polarized IR light can address the OSE of multiple exciton resonances simultaneously, providing the opportunity to directly compare the parameters of their polarization matrices.

**Figure 5:**
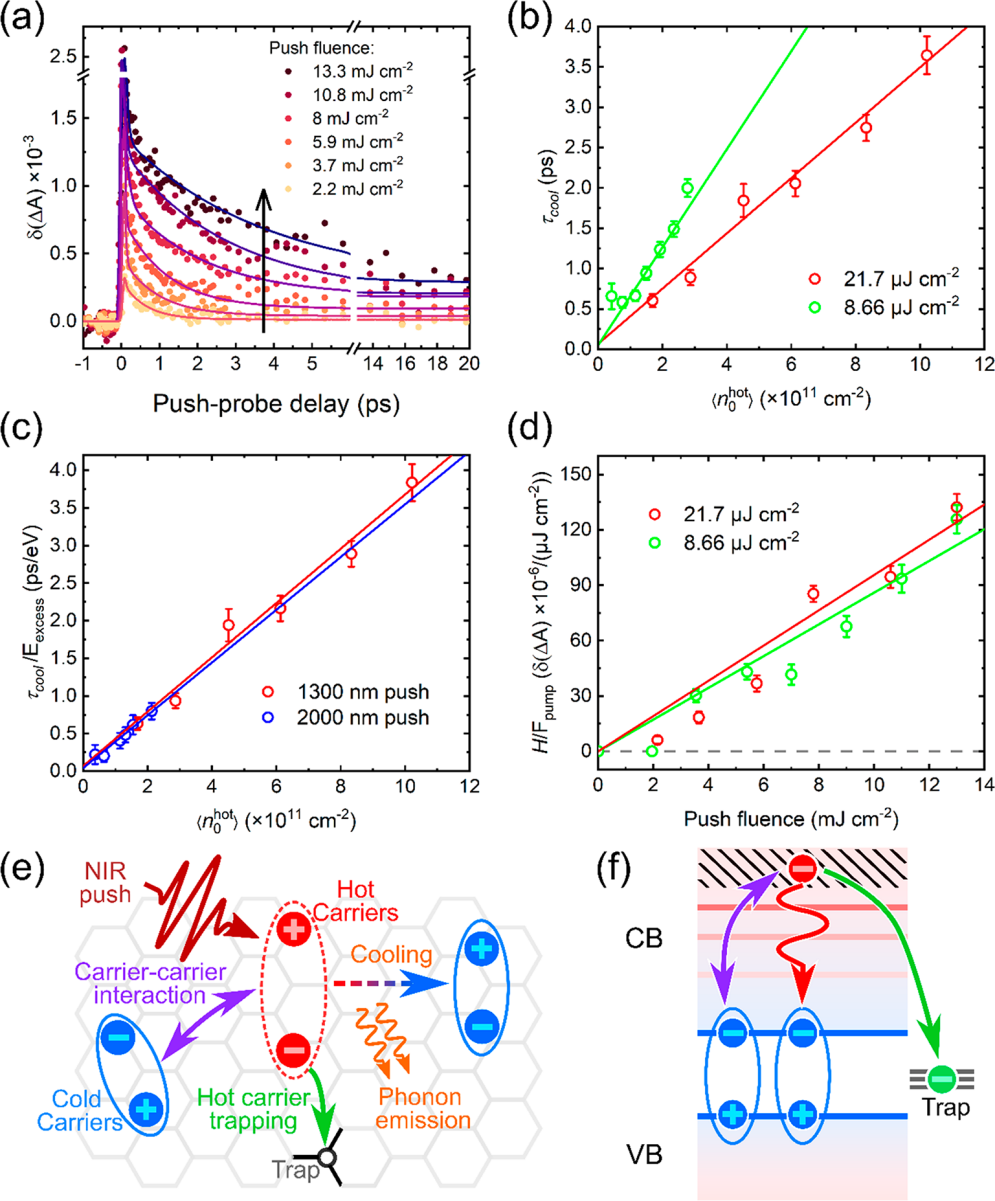
Hot carrier relaxation pathways in SL-WS_2_. (a) δ(Δ*A*) kinetics (PPP–PP) obtained from a 1300 nm push-fluence-dependent PPP measurement at a fixed pump fluence (21.7 μJ cm^–2^). The solid lines are fits by Gaussian-convoluted biexponential decay plus baseline. (b) Hot carrier cooling lifetime, τ_cool_, as a function of the initial hot carrier density, ⟨*n*_0_^*hot*^⟩, plotted for two different push fluences. (c) τ_cool_ per excess energy as a function of ⟨*n*_0_^*hot*^⟩ for 1300 and 2000 nm push, with the same pump fluence, 21.7 μJ cm^–2^. (d) Amplitude of the long-lived PPP signal, *H*, per pump fluence, *F*_pump_, for A exciton GSB as a function of push fluence. The solid lines in (b), (c), and (d) are linear fits. (e) and (f) Scheme and state diagram depicting the relaxation pathways of hot carriers in SL-WS_2_. The hot carriers are considered as free charges (dashed oval), and the cold carriers are considered as bound excitons at the band edge (solid oval). The relaxation pathways for hot carriers are shown as (1) phonon-assisted relaxation to the band-edge bound exciton, (2) carrier–carrier interactions, and (3) trapping. For simplicity we depict the hot electron relaxation in (f), but we expect the same effects on hot holes.

Based on previous studies,^26,57^ we assign the intermediate (∼ps) PPP decay component to the cooling of hot carriers to the band edge. As shown in Figures 5a and S5, the specific values of the hot carrier cooling times, τ_cool_, are determined by fitting the PPP transients to a biexponential decay plus a baseline, convoluted with the Gaussian response function, *G*(*t*):
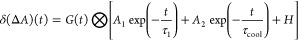
2where *A*_1_, τ_1_ and *A*_2_, τ_cool_ represent the amplitudes and decay times of the fast and slow decay components, and *H* is a constant value to represent the long-lived component. The decay time of the fast component, τ_1_, is fixed to 0.01 ps to numerically represent the instant OSE (see Figure S7), the cooling time *τ*_cool_ is a free parameter, and the amplitude of *H* reflects the density of trapped carriers as discussed below. The fitting results are presented in Figures 5a and S8 for different excitation conditions. Note that *H* is kept as zero for the δ(Δ*A*) kinetics with 2000 nm push, with fitting results presented in Figure S8b. Because a single free decay parameter appears sufficient to describe the full data set, we conclude that a two-state model, involving cold and hot excited states, can represent the key photophysics.^79^ It can be seen in Figure 5a that with the increase of the push fluence, the signal lifetime and the size of *H* increases. To analyze the HPB effect, the push fluence is converted into initial hot carrier density, ⟨*n*_0_^*hot*^⟩. This calculation is outlined in the Supporting Information, and the linear relationship between the push fluence and ⟨*n*_0_^*hot*^⟩ is confirmed by Figure S9. In addition, the linearity of the estimated initial hot carrier density, ⟨*n*_0_^*hot*^⟩, as a function of NIR push fluence in Figure S9 suggests that there is no multiphoton effect with the intense NIR push pulse.

Figure 5b shows the linear dependence of τ_cool_ on the initial hot carrier density after the push, ⟨*n*_0_^*hot*^⟩, with two pump fluences and at the same push wavelength (1300 nm). The linearity of the plot precludes many-body processes, such as Auger-type heating (another carrier is heated by the energy released from electron–hole recombination), under the experimental conditions in our measurements. The linear scaling of the cooling lifetime with the hot carrier density has been observed in other TMDs and other polar semiconductors by PP spectroscopy and assigned to the HPB effect.^44,57,58,80,81^ The HPB effect occurs when the cooling hot carriers deposit their excess energy into a limited pool of LO phonons.^32^ When the hot carrier density becomes too high, the excess energy cannot be efficiently dissipated into freely accessible phonons in the cold lattice, slowing the cooling down. This ultimately results in a linear increase of τ_cool_ as a function of ⟨*n*_0_^*hot*^⟩. The linear fit of τ_cool_ as a function of ⟨*n*_0_^*hot*^⟩ in Figure 5b provides a y-intercept value at ⟨*n*_0_^*hot*^⟩ = 0, corresponding to the “intrinsic” hot carrier cooling time of a single hot carrier in the absence of the HPB effect.^50^ The two different pump fluences with the 1300 nm (0.95 eV) push give the similar intercepts from the linear fits in Figure 5b, corresponding to a cooling rate of ∼18 ± 2.7 eV/ps. Comparing with previous studies on other materials, the cooling rate of polycrystalline SL-WS_2_ is much faster than halide perovskites (∼1 to 3 eV/ps) and other nanomaterials, such as CdSe quantum dots (sub-meV/ps) and graphene (a few eV/ps).^50,82,83^ Even though the cooling rate of SL-WS_2_ is fast, the HPB effect can efficiently prolong the cooling time to a few picoseconds when hot carrier density is increased.

In contrast to conventional PP spectroscopy, the PPP approach allows the cold carrier density to be varied independently from the hot carrier density to investigate the impact of electronic interactions on hot carrier cooling.^51^ In Figure 5b, we observe that with the increase of cold carrier density (i.e., pump fluence), the cooling time scale in SL-WS_2_ is shortened, and the dependence (slope) of τ_cool_ over ⟨*n*_0_^*hot*^⟩ is reduced.^50^ This signifies that the HPB effect is weakened by additional relaxation channels produced by the abundance of cold carriers that accept and share energy with the hot states. We have observed a similar acceleration of the cooling dynamics by cold carriers in our previous PPP studies on halide perovskites and nanocrystals.^51,79^

In our experiment, different push wavelengths provide different excess energies to the cold state. The cooling rate of the optically heated carriers, τ_cool_ per excess energy, *E*_*excess*_, as a function of ⟨*n*_0_^*hot*^⟩, is plotted in Figure 5c for 1300 and 2000 nm push at a fixed pump fluence. The linear fits of these two data sets can be interpolated to the same time constant (∼50 fs), which indicates that the intrinsic cooling rate for a single hot carrier is independent of its excess energy. The identical slopes of the two linear fits in Figure 5c suggests that the HPB effect in SL-WS_2_ is linearly dependent on the excess energy of the hot carriers when the cold carrier density is fixed. Taking the thickness of SL-WS_2_ as ∼0.86 nm from AFM (Figure S1), the slope of the linear fit with the 2000 nm push (0.62 eV excess energy) gives a carrier cooling rate of ∼0.17 ± 0.02 ps per 10^18^ cm^–3^, which is up to four times lower than that in halide perovskites thin films (from ∼0.25 to 1 ps per 10^18^ cm^–3^) measured with the same excess energy.^50^ This indicates that the HPB effect in the SL-WS_2_ is much weaker than in halide perovskite materials,^50^ which might be caused by the pronounced excitonic nature of the excited states and their extreme quantum confinement in the atomically thin TMD layer.^84,85^

We now turn to the third component of the PPP transients in Figure 3e. In the case of the 1300 nm push, this component appears to emerge within the time scale of intraband relaxation but persists beyond the lifetime of the hot carriers, which suggests that not all of the optically excited hot carriers cool to the band edge. Interestingly, this long-lived (>20 ps) component represented by the constant *H* value falls to zero when a lower-energy 2000 nm push is used, as seen in Figures 3e and S4b. Both push photon energies (0.95 eV for 1300 nm, and 0.62 eV for 2000 nm) are greater than the exciton binding energy in SL-WS_2_ (∼0.3 eV).^15,86^ From this, we infer that exciton dissociation is not responsible for the long-lived signal.

An alternative explanation for the long-lived signal is that the higher-energy push drives the exciton over the energy barrier (∼0.88 eV) between the K (A exciton) valley to the band nesting region (C exciton). However, the massless push photons cannot provide momentum to the carriers to enable scattering between valleys in the Brillouin zone. Even if this intervalley scattering were to occur, we expect that carriers scattered to other valleys should eventually flow back to the band edge of the K (or K′) valley within a few hundred femtoseconds rather than tens of picoseconds.^44^ These factors suggest that the long-lived signal is also not likely caused by an intervalley transition.

The long-lived component, *H*, is normalized by the respective pump fluence, *F*_*pump*_, in Figure 5d. The values of *H*/*F*_*pump*_ are very similar across the range of push fluences used, which strongly indicates that the long-lived component in the δ(Δ*A*) kinetics with 1300 nm push is dominated by hot carriers rather than cold carriers. Furthermore, the linearity of *H*/*F*_*pump*_ with the push fluence suggests that the long-lived component is not caused by many-body interactions.

Taking all the evidence above, we propose that the impartial recovery of the GSB upon pushing at 1300 nm is the result of hot carrier trapping. The trap states can originate from the defects of the polycrystalline SL-WS_2_ deposited by CVD, such as S vacancies, nucleation centers, and grain boundaries, which are known to create additional energy levels that act as charge capture sites within the band gap.^5,87,88^ The full recovery of the GSB at 2000 nm (0.62 eV) push in Figure S8b demonstrates that there is an energy barrier in the hot carrier trapping process. Similar trapping phenomena have been observed in other semiconductors by PPP, including halide perovskites and quantum dots,^53,56^ and have been used to rationalize the low PL efficiencies of these materials after high-energy excitation.^56^ The weaker emission from highly excited SL TMDs as opposed to their resonantly excited counterparts has also been reported but have been ascribed to charge separation in the Brillouin zone in the band nesting region.^37^ Because defects in these materials are invariably produced by CVD, our results suggest that hot carrier trapping can also contribute to the reduced PL efficiency in TMDs after high energy photoexcitation.

Finally, it is worth remarking on the effect of the sample preparation on the observed results. In contrast to mechanically exfoliated SL-TMD flakes, CVD-grown polycrystalline TMD systems have more intrinsic defects such as chalcogenide atom vacancies and grain boundaries.^89^ These defects can introduce trap states for carriers, which are potentially responsible for the hot carrier trapping observed herein. Further prospective studies, involving controlled deposition and encapsulation of the mechanically exfoliated SL-TMD flakes, could help to explore this possibility further. These strategies have been employed to control the defect density and exciton binding energy (via Coulomb screening) in TMD materials.^15,86,90^

To consolidate all of our findings, and paint a complete picture of the fate of hot carriers in a benchmark TMD based on experimental observations, we summarize the hot carrier relaxation pathways for SL-WS_2_ in Figure 5e,f. The free hot carriers cool down from the hot state to the band edge to form cold bound excitons through two main pathways: (1) phonon-assisted pathway by that can prolong the cooling time via the HPB effect and (2) carrier-assisted pathway by carrier–carrier interactions to accelerate the cooling process. A portion of hot carriers can also be trapped when their excess energy is sufficiently high.

## Conclusion

This work showcases PPP spectroscopy as an approach to characterize the dynamics and fate of hot carriers in a benchmark atomically thin semiconductor, SL-WS_2_. By carefully controlling the excitation conditions, we demonstrate the existence of a HPB below the Mott transition, which manifests as a slowing of the carrier cooling with increasing hot carrier density. We find the intrinsic cooling time of an isolated hot carrier in SL-WS_2_ to be substantially faster than other emerging high-performance semiconductors. However, the efficient HPB effect can dramatically prolong this cooling time to a few picoseconds. In contrast to previous studies of TMDs, we observe an additional hot carrier cooling mechanism via carrier–carrier interactions, as evidenced by a suppression of the HPB in the presence of cold carriers. Our three-pulse approach also unveils that highly excited hot carriers can be trapped during cooling, which prevents the formation of band-edge excitons that are chiefly responsible for light emission. As well as disclosing information about fundamental carrier-phonon and carrier–carrier interactions and their interplay in TMDs, our findings bring insight into the mechanisms and time scales of performance-limiting processes in TMD-based optoelectronics and could have implications for other applications in high-speed switching, spintronic and quantum technologies.

## Methods

A fully covered polycrystalline SL-WS_2_ on quartz substrate (1 cm × 1 cm) produced via low-pressure chemical vapor deposition was commercially procured (CVD-TSF-WS2-QZ, 2D Semiconductors, United States) and measured without further modification. The UV–vis spectrum was measured with a Shimadzu UV-2600 UV–vis spectrophotometer. Raman and PL spectra were both collected by a Reminder lab Raman with a 50× objective lens and 532 nm laser excitation in an incident power of 0.16 mW at the same spot. The SL-WS_2_ thickness was roughly determined by a Keysight 5500 SPM AFM (Keysight Technologies) with a point-probe (Silicon-SPM-Sensor, PPP-NCLR-50, Nanosensors) operating in a noncontact mode. AFM scan was performed at a 10 μm × 10 μm area at a scanning rate of 0.40 Hz. PPP spectroscopy was adapted from a commercial transient absorption spectrometer (HELIOS, Ultrafast Systems). Details for the PPP spectroscopy are given in the Supporting Information.

## Supporting Information Available

Sample characterization by AFM, layout of the PPP spectroscopy setup, amplitude of A exciton GSB at different pump–probe delay times, TA of SL-WS_2_ with NIR probe, line shape analysis for push effect, carrier temperature analysis using Maxwell–Boltzmann distribution, fits of PPP kinetics, and estimation of the hot carrier density (PDF)
