# Supplementary material for: Hot Carrier Cooling and Trapping in Atomically Thin WS2 Probed by Three-Pulse Femtosecond Spectroscopy
Source: ACS Nano. 2023 Mar 20;17(7):6330–40. doi: 10.1021/acsnano.2c10479 (PMC10100566; doi:10.1021/acsnano.2c10479)
Supplement: Supplementary file 1 — nn2c10479_si_001.pdf [file nn2c10479_si_001.pdf]

Supporting Information for

Hot Carrier Cooling and Trapping in Atomically-Thin WS_2 Probed by Three-Pulse Femtosecond Spectroscopy

Tong Wang,[1] Thomas R. Hopper,[1,2] Navendu Mondal,[1] Sihui Liu,[1] Chengning Yao,[1] Xijia Zheng,[1] Felice Torrisi,[1,3] Artem A. Bakulin[1*]*

[1]Department of Chemistry and Centre for Processable Electronics, Imperial College London, London W12 0BZ, United Kingdom

[2]Department of Materials Science and Engineering, Stanford University, Stanford, California 94305, United States

[3]Dipartimento di Fisica e Astronomia, Universita' di Catania & CNR-IMM (Catania Universita'), Via S. Sofia 64, 95123, Catania, Italy

* Corresponding author: trh@stanford.edu, a.bakulin@imperial.ac.uk;

Determination of film thickness:

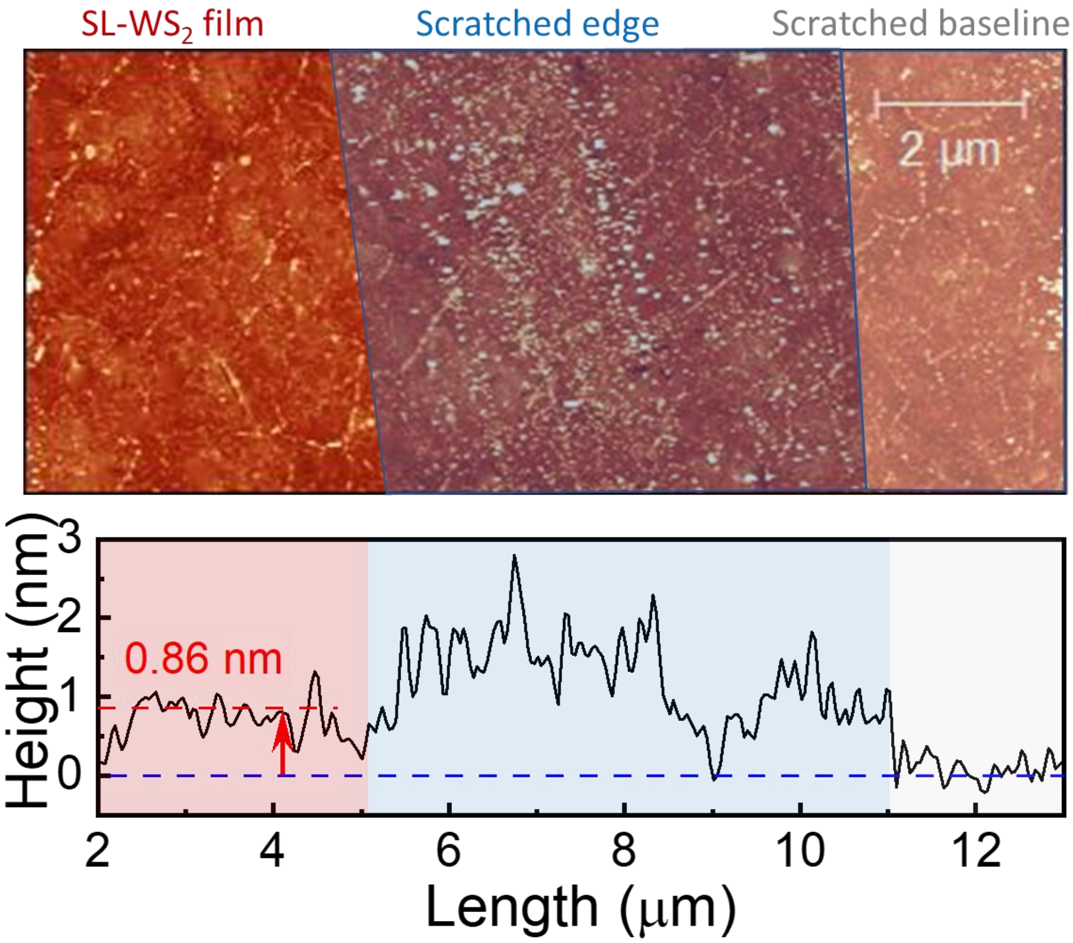

Figure S1. AFM image of SL-WS$_2$. *The AFM tip height profile demonstrates the thickness of the SL-WS$_2$ as a function of length from the scratched edge. The scale bar is 2 μm.*

The SL-WS$_2$ film fully covering the substrate was scratched by a sharp needle in the central part of the sample, leaving the exposed quartz substrate as a baseline for the AFM measurement. This blank substrate area can be seen at the right of the top panel of Figure S1, with the scratched edge in the middle region, and the SL-WS$_2$ film in the left region. The scratched edge region is ignored for the determination of the sample thickness. The real thickness of SL-WS$_2$ is determined by the average height of left region (dashed red line), relative to the baseline in the right region (dashed blue line). The 0.86 nm thickness further confirms the SL character of the sample in our measurement.[1,2] The AFM measurement was performed after all optical measurements.

Layout of setup:

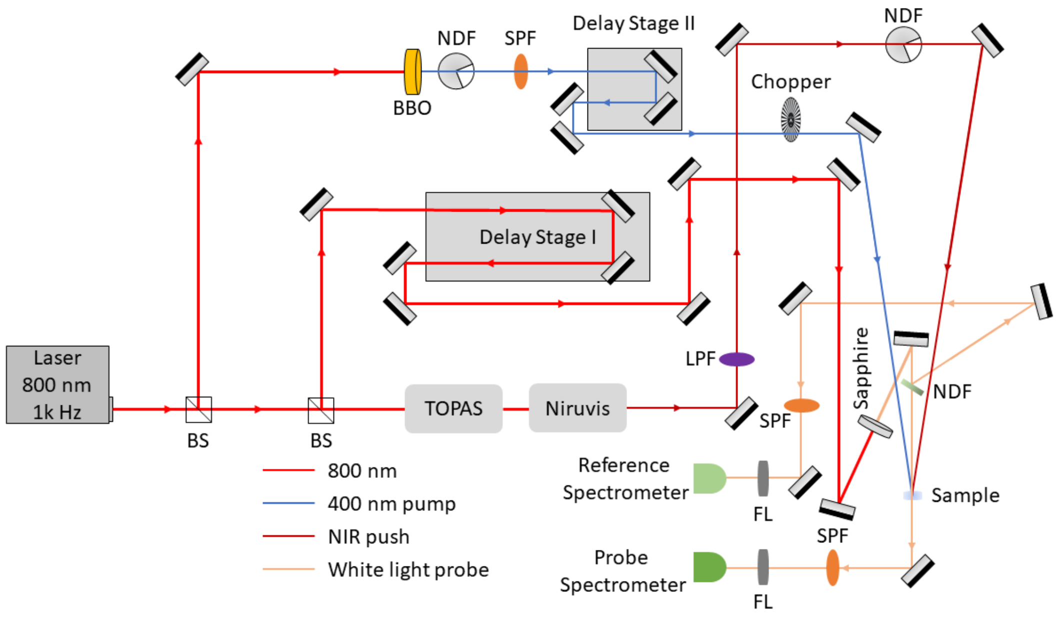

***Figure S2**. **Layout of the pump-push-probe and transient absorption setup.** BS: beam splitter; BBO: beta barium borate crystal; SPF: short-pass filter; LPF: long-pass filter; FL: focus lens; NDF: neutral density filter; TOPAS: optical parametric amplifier; Niruvis: frequency mixer.*

The ultrafast experiments are based on a commercial Helios transient absorption spectroscopy setup (Spectra Physics, Newport Corp.). Ultrafast 800 nm (1 kHz, <100 fs pulse duration) laser pulses were generated by a Ti:sapphire regenerative amplifier (Solstice, Spectra Physics, Newport Corp.). This fundamental output is split into three portions. The first portion is directed to a beta barium borate (BBO) crystal for the 400 nm pump pulse via second-harmonic generation. The second portion is fed into an optical parametric amplifier (TOPAS Prime, Spectra-Physics) and a frequency mixer (Niruvis, Light Conversion) for the NIR push pulse (1300 or 2000 nm, signal or idler). The remaining 800 nm pulses are fed into a sapphire crystal to generate the broadband white light probe (450 – 750 nm), or a YAG crystal to generate the broadband NIR probe (900 – 1400 nm).

In our setup, the push beam is fixed in path length, and the probe and pump are delayed by automated and manual mechanical stages (Delay Stage I and II, respectively). The three

beams are focused onto the sample at the same spot (diameter ~0.5 mm), and the transmitted probe is collected by a CCD spectrometer. To mitigate shot-to-shot noise, we use another CCD spectrometer to measure fluctuations in a reference beam that is split off from the probe before it hits the sample by a neutral density filter. A mechanical chopper in the pump beam is modulated at 500 Hz to block every other pulse and relay the TA signal.

Amplitude of A exciton GSB:

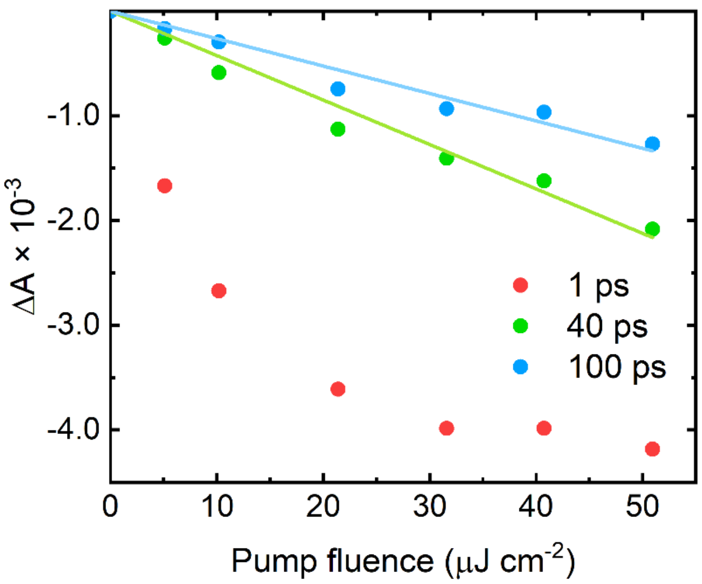

Figure S3. TA amplitude of the A exciton GSB for different pump-probe delays as a function of the 400 nm pump fluence. *The solid lines are linear fits of amplitude of A exciton GSB at 40 and 100 ps.*

The apparent nonlinear relationship of the A exciton GSB amplitude with the pump fluence at 1 ps suggests that many-body interaction happens at very early time after 400 nm excitation. At longer time delays (>40 ps), the amplitude of A exciton GSB is linear with pump fluence, indicating eventual mono-molecular recombination following 400 nm excitation.

TA with NIR probe:

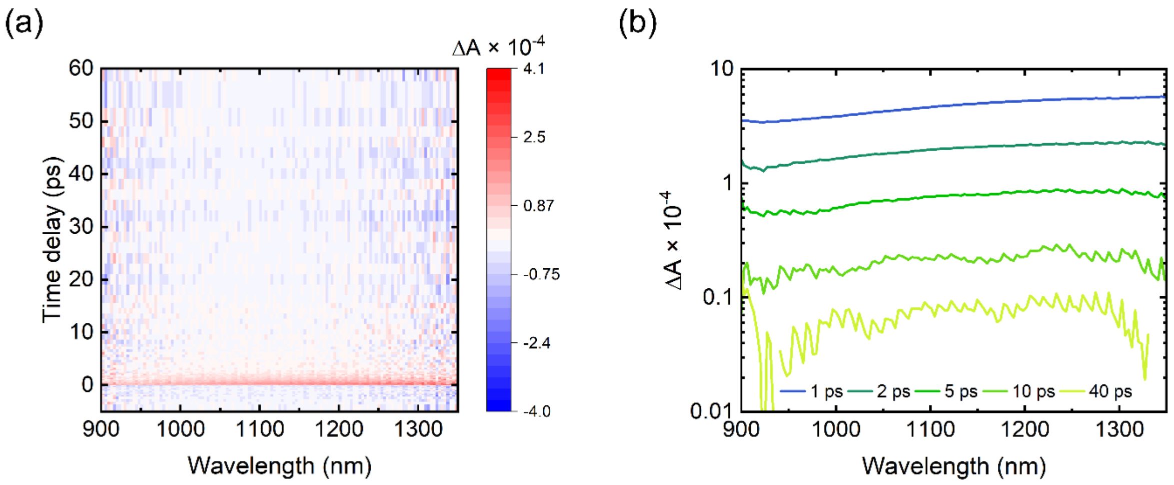

Figure S4. Exciton-to-continuum transition in SL-WS$_2$. *(a) TA heatmap and (b) corresponding TA spectra at different time slices for the monolayer WS$_2$. A 400 nm pump (50.9 μJ cm^{-2}) and NIR probe were used.*

The featureless broadband absorption of the NIR probe without any sharp excitonic features confirms that the transition of the A exciton from the lowest-energy 1S ‘cold’ state to the ‘hot’ continuum state after the NIR push. Singular value decomposition shows the dataset has only one dominant component.

Lineshape analysis for A exciton at the arrivial of NIR push pulse:

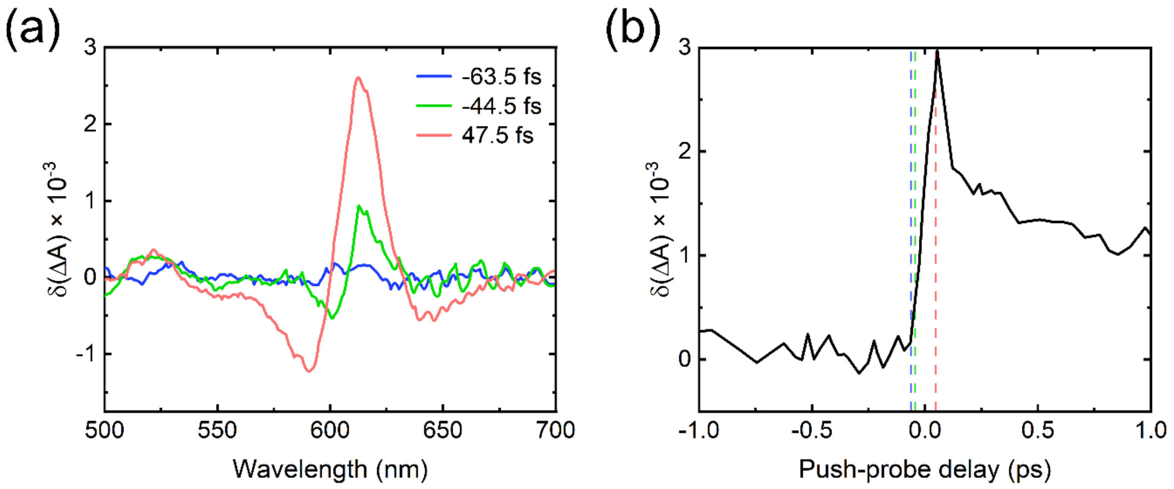

Figure S5. Negative time-delay data for the A exciton. *(a) $\delta(\Delta A)$ spectrum at negative push-probe delay times. The $\delta(\Delta A)$ spectrum is obtained by subtracting the PP TAS spectrum (1300 nm push off) from the PPP TAS (1300 nm push on, fluence: 10.6 mJ cm^{-2}) spectrum under the same excitation conditions. (b) $\delta(\Delta A)$ transient for the A exciton GSB, with time slices for the corresponding spectra in (a). The blue, green and red dashed lines represent push-probe delays at -63.5, -44.5 and 47.5 fs, respectively.*

The effects of static and dynamic disorder lead to the ultrafast dephasing times and the suppression of the OSE at negative delays (Figure S5), in contrast to the OSE observed in some well-controlled quantum well systems.[3–5] Moreover, given the similarity between the observed OSE spectra and the first derivative of the absorption spectrum, and absence of additional oscillatory features in the spectral response, we conclude that the interaction occurs in the weak excitation limit.[3,6]

Maxwell-Boltzmann distribution for carrier temperature:

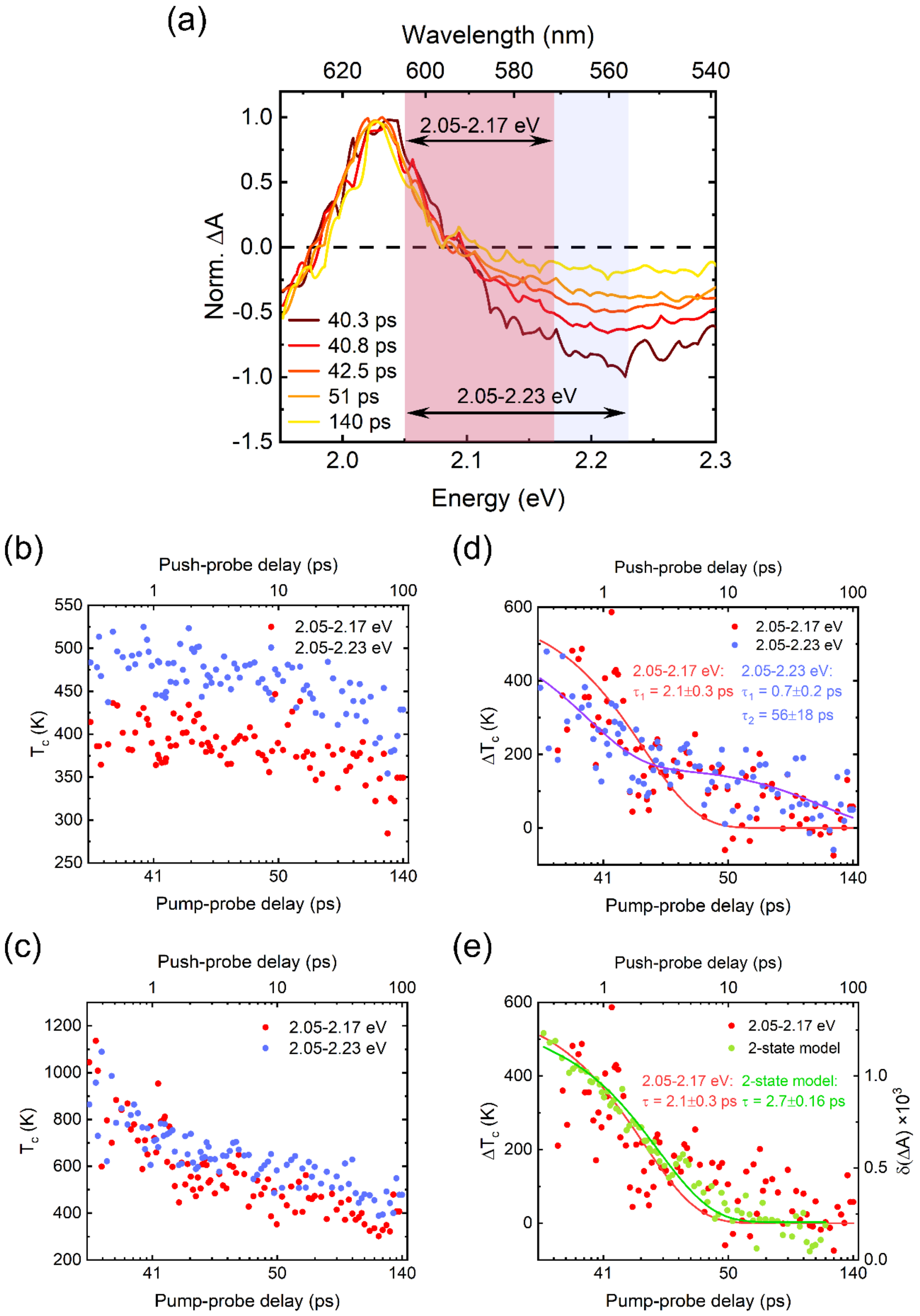

Figure S6. Carrier temperature estimated by Maxwell-Boltzmann distribution. *(a) Normalised TA spectrum after NIR push (400 nm pump, 21.7 μJ cm^{-2}; 1300 nm push,10.6 mJ cm^{-2}). The carrier temperature is extracted for two different fitting windows: 2.05-2.17 eV (red) and 2.05-2.23 eV (blue). (b) and (c) plot these extracted carrier temperatures for the PP and PPP measurements respectively (same pump excitation conditions). The data is shown after 40.3 ps of pump-probe delay (0 ps of push-probe delay) as a comparison and avoiding the complexity of OSE caused by the NIR push pulse. (d) compares the temperature decay kinetics with different fitting windows. (e) compares the kinetics fitted by Maxwell-Boltzmann distribution (2.05-2.17 eV) and the kinetics fitted by the 2-state model.*

A Maxwell-Boltzmann distribution was implemented to estimate the carrier temperature after NIR push reheating. Figure S6 shows the estimated carrier temperature with 400 nm pump (21.7 μJ cm^{-2}) and 1300 nm push (10.6 mJ cm^{-2}). In previous reports, the high energy region of the GSB is used to estimate of carrier temperature.[7–10] We extracted the carrier temperature from two spectral windows: 2.05-2.17 eV (in red) and 2.05-2.23 eV (in blue). As shown in Figure S6 b,c, the absolute estimated carrier temperature is dependent on the selected fitting window for both PP and PPP measurements. Figure S6d plots the push-induced carrier temperature difference obtained from the subtraction of the PPP dataset (push on) from the PP dataset (push off). The cooling dynamics vary dramatically with the fitting window. With the narrow window (2.05-2.17 eV) the cooling dynamics can be described by a monoexponential decay, but with the wider window (2.05-2.23 eV), a biexponential decay function is needed. The dynamics and timescales of the monoexponential fit are similar to those obtained from the two-state model presented in the main text, with time constants of 2.1 ± 0.16 ps and 2.7 ± 0.16 ps, respectively. However, due to the ambiguity in carrier temperatures, we use the two-state model as a framework to describe our results, which separates the carriers into 'hot' (above band-edge) and cold (band-edge) without specifying the carrier temperature.

PPP transients:

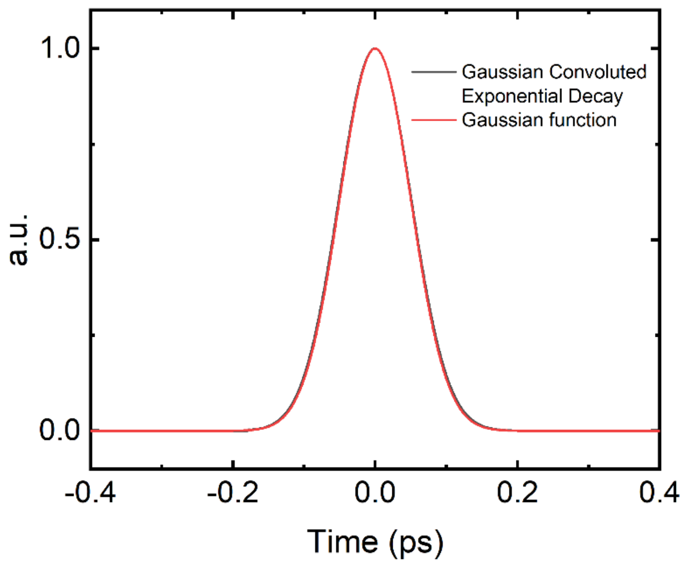

Figure S7. Comparison of Gaussian function with and without convoluted exponential decay. *Red: Gaussian function without convolution and fixed σ value (0.05 ps.) Black: same Gaussian function convoluted with an exponential decay (0.01 ps time constant).*

Figure S7 shows that when the time constant for the exponential decay is sufficiently short (i.e. 0.01 ps), a Gaussian convoluted exponential decay can represent the Gaussian function numerically. The optical Stark effect should follow a Gaussian instrumental response function in the time domain,[11,12] therefore, the fast component in Equation 2 in the main text is fixed at 0.01 ps to represent the coherent optical Stark effect.

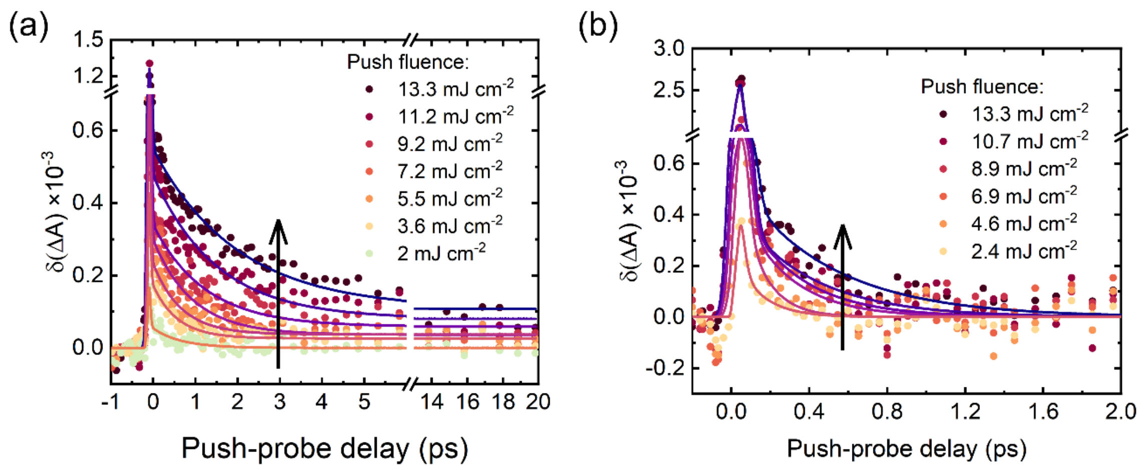

***Figure S8. Push fluence dependence**. Push-induced kinetics obtained by subtracting the PPP transients (push on) from the PP transients (push off) to give a $\delta(\Delta A)$ transient. Transients obtained from (a) 1300 nm push, and a 400 nm pump fluence at 8.66 $\mu J\ cm^{-2}$; and (b) 2000 nm push, and a 400 nm pump fluence at 21.7 $\mu J\ cm^{-2}$. The solid lines are fits by Gaussian-convoluted biexponential decay plus baseline.*

Estimation of initial hot carrier density:

The initial carrier density excited by the 400 nm pump, n_0^{total}, is calculated by Equation S1:

$$n_0^{total} = A_{400} \frac{F}{3.1\ eV} \qquad \text{(Equ. S1)}$$

where A_{400} is the absorbance of SL-WS_2 at 400 nm which obtained from the UV-vis absorption spectrum in Figure 1a, and *F* is the incident 400 nm pump fluence (in units of eV cm^{-2}).

Herein, we describe two methods to estimate the initial hot carrier density, $\langle \mathrm{n}_0^{\mathrm{hot}} \rangle$.

Method 1: The cold carrier density just before the push pulse, n_0^{cold}, can be described by Equation S2. $GSB_{39.8\ ps}$ is the amplitude of the A exciton GSB signal at 39.8 ps pump-probe delay, and GSB_{max} is the maximum of the GSB signal. We assume that the A exciton GSB signal at 40 ps is the same as the GSB signal at 39.8 ps.

$$n_0^{cold} = n_0^{total} \frac{GSB_{39.8\ ps}}{GSB_{max}} \qquad \text{(Equ. S2)}$$

As shown in Figure 5a, the optical Stark effect shows up in the A exciton GSB signal at the arrival of the push pulse. Therefore, to avoid the interference by the optical Stark effect, we used the amplitude difference between the A exciton GSB signal at 40.2 ps (hot) and at 39.8 ps (cold) to represent the initial hot carrier density, $\langle n_0^{hot} \rangle$. By using the Equation S3 and substituting n_0^{cold} from Equation S2, we can get the approximate value of $\langle n_0^{hot} \rangle$ with different push fluences. The estimated $\langle n_0^{hot} \rangle$ as the function of push fluence is shown in Figure S9.

$$\langle n_0^{hot} \rangle = n_0^{cold} \frac{GSB_{40.2\ ps} - GSB_{39.8\ ps}}{GSB_{39.8\ ps}} = n_0^{total} \frac{GSB_{40.2\ ps} - GSB_{39.8\ ps}}{GSB_{max}} \qquad \text{(Equ. S3)}$$

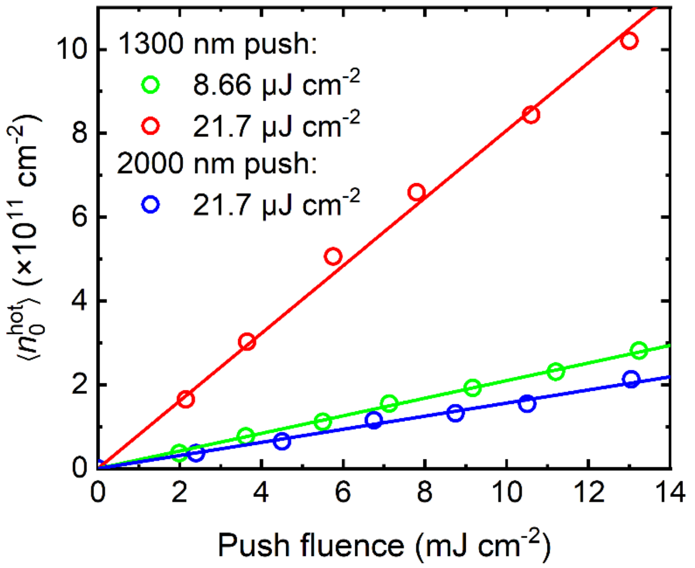

Figure S9. Effect of push on hot carrier density. *Linear relationship of initial hot carrier density, $\langle n_0^{hot} \rangle$, with push fluence. The solid lines are linear fits.*

Method 2: The initial carrier density, $\mathrm{n}_0^{\mathrm{total}}$, can be substituted into the multi-exponential decay fit function of A exciton GSB kinetics obtained from PP measurements to get the value of $\mathrm{n}_0^{\mathrm{cold}}$ at 40 ps (pump-probe decay). Then, $\langle \mathrm{n}_0^{\mathrm{hot}} \rangle$ can be calculated by substitute the value of $\mathrm{n}_0^{\mathrm{cold}}$ into the Equation S3 (the intermediate step).

The estimated $\langle \mathrm{n}_0^{\mathrm{hot}} \rangle$ with 2 methods are shown in Figure S10. Although this estimation method and the assumptions may not reveal the exact value of $\langle n_0^{hot} \rangle$, by using the same data analysis protocol for all datasets, the trend of cooling time we present in the main text can still represent the hot carrier cooling behaviour of the monolayer WS_2.

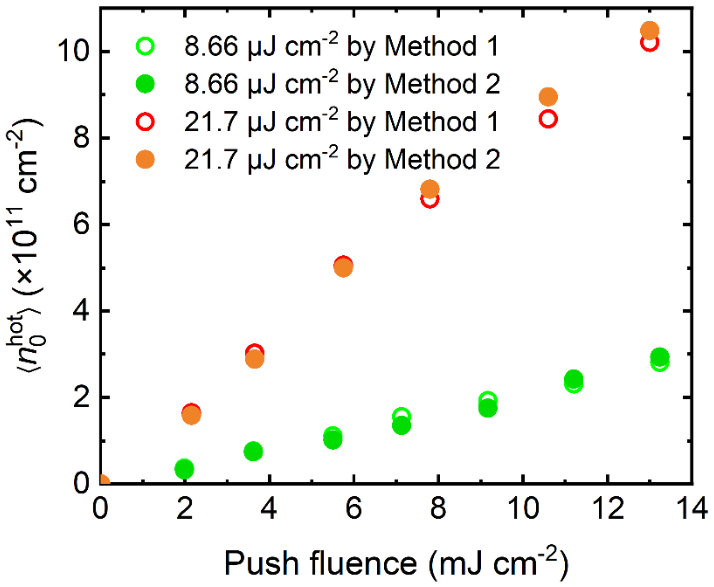

Figure S10. **$\langle n_0^{hot} \rangle$ *estimation by two different methods.*** *The open circle is the estimated $\langle n_0^{hot} \rangle$ by the method described in SI, Method 1. The solid circle is the estimated $\langle n_0^{hot} \rangle$ by substituting the absorbed photon density into the A exciton GSB decay function, Method 2.*

References:

(1) Zhang, Y.; Zhang, Y.; Ji, Q.; Ju, J.; Yuan, H.; Shi, J.; Gao, T.; Ma, D.; Liu, M.; Chen, Y.; Song, X.; Hwang, H. Y.; Cui, Y.; Liu, Z. Controlled Growth of High-Quality Monolayer WS2 Layers on Sapphire and Imaging Its Grain Boundary. *ACS Nano* **2013**, *7*, 8963–8971.

(2) Taghavi, N. S.; Gant, P.; Huang, P.; Niehues, I.; Schmidt, R.; Michaelis de Vasconcellos, S.; Bratschitsch, R.; García-Hernández, M.; Frisenda, R.; Castellanos-Gomez, A. Thickness Determination of MoS2, MoSe2, WS2 and WSe2 on Transparent Stamps Used for Deterministic Transfer of 2D Materials. *Nano Res.* **2019**, *12*, 1691–1695.

(3) Unold, T.; Mueller, K.; Lienau, C.; Elsaesser, T.; Wieck, A. D. Optical Stark Effect in a Quantum Dot: Ultrafast Control of Single Exciton Polarizations. *Phys. Rev. Lett.* **2004**, *92*, 1–4.

(4) Guenther, T.; Lienau, C.; Elsaesser, T.; Glanemann, M.; Axt, V. M.; Kuhn, T.; Eshlaghi, S.; Wieck, A. D. Coherent Nonlinear Optical Response of Single Quantum Dots Studied by Ultrafast Near-Field Spectroscopy. *Phys. Rev. Lett.* **2002**, *89*, 057401.

(5) Saba, M.; Quochi, F.; Ciuti, C.; Martin, D.; Staehli, J.-L.; Deveaud, B.; Mura, A.; Bongiovanni, G. Direct Observation of the Excitonic Ac Stark Splitting in a Quantum Well. *Phys. Rev. B* **2000**, *62*, R16322–R16325.

(6) Mysyrowicz, A.; Hulin, D.; Antonetti, A.; Migus, A.; Masselink, W. T.; Morkoç, H. “Dressed Excitons” in a Multiple-Quantum-Well Structure: Evidence for an Optical Stark Effect with Femtosecond Response Time. *Phys. Rev. Lett.* **1986**, *56*, 2748–2751.

(7) Li, M.; Bhaumik, S.; Goh, T. W.; Kumar, M. S.; Yantara, N.; Grätzel, M.; Mhaisalkar, S.; Mathews, N.; Sum, T. C. Slow Cooling and Highly Efficient Extraction of Hot Carriers in Colloidal Perovskite Nanocrystals. *Nat. Commun.* **2017**, *8*, 3–12.

(8) Price, M. B.; Butkus, J.; Jellicoe, T. C.; Sadhanala, A.; Briane, A.; Halpert, J. E.; Broch, K.; Hodgkiss, J. M.; Friend, R. H.; Deschler, F. Hot-Carrier Cooling and Photoinduced Refractive Index Changes in Organic-Inorganic Lead Halide Perovskites. *Nat. Commun.* **2015**, *6*, 1–8.

(9) Lim, J. W. M.; Giovanni, D.; Righetto, M.; Feng, M.; Mhaisalkar, S. G.; Mathews, N.; Sum, T. C. Hot Carriers in Halide Perovskites: How Hot Truly? *J. Phys. Chem. Lett.* **2020**, *11*, 2743–2750.

(10) Yin, J.; Naphade, R.; Maity, P.; Gutiérrez-Arzaluz, L.; Almalawi, D.; Roqan, I. S.; Brédas, J. L.; Bakr, O. M.; Mohammed, O. F. Manipulation of Hot Carrier Cooling Dynamics in Two-Dimensional Dion–Jacobson Hybrid Perovskites via Rashba Band Splitting. *Nat. Commun.* **2021**, *12*, 1–9.

(11) Sie, E. J.; McLver, J. W.; Lee, Y. H.; Fu, L.; Kong, J.; Gedik, N. Valley-Selective Optical Stark Effect in Monolayer WS2. *Nat. Mater.* **2015**, *14*, 290–294.

(12) Cunningham, P. D.; Hanbicki, A. T.; Reinecke, T. L.; McCreary, K. M.; Jonker, B. T. Resonant Optical Stark Effect in Monolayer WS2. *Nat. Commun.* **2019**, *10*, 5539.
